# The Long Non-Coding *HOTAIR* Is Modulated by Cyclic Stretch and WNT/β-CATENIN in Human Aortic Valve Cells and Is a Novel Repressor of Calcification Genes

**DOI:** 10.1371/journal.pone.0096577

**Published:** 2014-05-01

**Authors:** Katrina Carrion, Jeffrey Dyo, Vishal Patel, Roman Sasik, Salah A. Mohamed, Gary Hardiman, Vishal Nigam

**Affiliations:** 1 Department of Pediatrics (Cardiology), University of California San Diego, La Jolla, California, United States of America; 2 Department of Medicine, University of California San Diego, La Jolla, California, United States of America; 3 Department of Cardiac Surgery, University Clinic of Schleswig-Holstein, Campus Luebeck, Luebeck, Germany; 4 Department of Medicine, Medical University of South Carolina, Charleston, South Carolina, United States of America; 5 Computational Science Research Center & Biomedical Informatics Research Center San Diego State University, San Diego, California, United States of America; 6 Rady Children’s Hospital San Diego, San Diego, California, United States of America; University of California San Diego, United States of America

## Abstract

Aortic valve calcification is a significant and serious clinical problem for which there are no effective medical treatments. Individuals born with bicuspid aortic valves, 1–2% of the population, are at the highest risk of developing aortic valve calcification. Aortic valve calcification involves increased expression of calcification and inflammatory genes. Bicuspid aortic valve leaflets experience increased biomechanical strain as compared to normal tricuspid aortic valves. The molecular pathogenesis involved in the calcification of BAVs are not well understood, especially the molecular response to mechanical stretch. *HOTAIR* is a long non-coding RNA (lncRNA) that has been implicated with cancer but has not been studied in cardiac disease. We have found that *HOTAIR* levels are decreased in BAVs and in human aortic interstitial cells (AVICs) exposed to cyclic stretch. Reducing *HOTAIR* levels via siRNA in AVICs results in increased expression of calcification genes. Our data suggest that **β**-CATENIN is a stretch responsive signaling pathway that represses *HOTAIR*. This is the first report demonstrating that *HOTAIR* is mechanoresponsive and repressed by WNT **β**-CATENIN signaling. These findings provide novel evidence that *HOTAIR* is involved in aortic valve calcification.

## Introduction

Aortic valve calcification/stenosis is the third leading cause of adult heart disease [1] and the most common form of acquired valvular disease in developed countries [2]. The risk factor most closely linked to calcific aortic stenosis is bicuspid aortic valve (BAV) [2], [3], [4], [5] since 64% of calcified aortic valves have BAV morphology [6]. While the role of biomechanical stretch is important to a number of different cell types in the body, it is especially important in the cardiovascular system since cells in the heart and arteries are exposed to stretch every time the heart beats. Calcification of the bicuspid aortic valve is an example of a cardiac disease in which increased biomechanical stretch is associated with disease. Individuals born with bicuspid aortic valve, which occurs in 1–2% of the population [2], [4], [5], have two aortic valve leaflets instead of the typical three leaflets. BAV leaflets experience increased stretch as compared to normal tricuspid aortic valves (TAVs) with every heartbeat [7], [8]. As a result of the role of biomechanical stretch in BAVs, we sought to examine the molecular responses of human aortic valve interstitial cells (AVICs) upon exposure to cyclic stretch.

While it is clear that osteogenic [9], [10], [11] and inflammatory [12], [13], [14], [15], [16], [17], [18] pathways are involved in aortic valve calcification, the mechanism by which these pathways are activated in BAVs has not been elucidated. We have previously found that microRNAs, a class of short non-coding RNAs, altered in calcified BAVs can modulate calcification related genes [19]. As a result of our previous work [8], [20], we were interested in identifying stretch responsive non-coding RNAs that would increase expression of calcification associated genes in aortic valve cells. We focused our efforts on examining the role of *HOTAIR* in AVIC response to stretch given that *HOTAIR* has been shown to play roles in human disease [21], [22], [23], [24], [25]. However, *HOTAIR* has not been reported as having a role in cardiac disease. *HOTAIR*, a 2.2 kb long non-coding RNA (lncRNA), was initially described as modulating HOX gene expression [26]. *HOTAIR* has been found to down regulate target genes by helping recruit the Polycomb Repression Complex 2 (PRC2) to target genes [21], [26], [27]. PRC2, which includes EZH2 and SUZ12, trimethylates H3 lysine K27 (H3K27me3) resulting in epigenetic silencing.

In this report we demonstrated that human AVICs exposed to stretch have lower levels of *HOTAIR*. BAV leaflets have a trend towards lower *HOTAIR* levels. Targeting *HOTAIR* via a siRNA resulted in higher expression of two osteogenic genes, *ALPL* [9], [28], [29] and *BMP2* [10], [30], that are required for calcification of AVICs [11], [31]. Additionally, microarray analysis of *HOTAIR* siRNA treated AVICs showed activation of additional calcification related genes and pathways. Since the WNT/**β**-CATENIN signaling pathway has been implicated in aortic valve calcification [32], [33], [34], [35] and other calcification [36], [37], [38], [39], [40] processes, we sought to determine if WNT/**β**-CATENIN was involved in this novel modulation of *HOTAIR*. Specifically, **β**-CATENIN is found at higher levels in calcified aortic valves [32]. Cyclic stretch increased the expression of ***β*** *-CATENIN* and **β**-CATENIN target genes, thereby implicating WNT/**β**-CATENIN signaling in the AVIC response to biomechanical stimuli. Treatment with WNT agonist results in increased *ALPL* and *BMP2* expression while repressing *HOTAIR*. These findings suggest that *HOTAIR* plays a role in repressing calcification-associated genes. A potential regulatory mechanism for *HOTAIR* repression in stretched AVICs would involved increased **β**-CATENIN signaling in turn repressing *HOTAIR*.

To our knowledge, this is the first evidence that *HOTAIR* is mechanoresponsive and repressed by WNT/**β**-CATENIN signaling. The linkages of a lncRNA to aortic valve disease and to biomechanical response pathways are novel findings. These data support the hypothesis that AVICs exposed to increased biomechanical stressors can activate calcification pathways associated with aortic valve calcification.

### Ethics Statement

The bicuspid aortic valves were collected under a protocol approved by the IRB at University Clinic of Schleswig-Holstein, Campus Luebeck, Luebeck, Germany. Written consent was obtained under this protocol. The control valves were obtained from organ donors whose hearts could not be used for transplantation. Written consent for the use of tissue for research was obtained from the next of kin by Lifesharing, a donate life organization (www.lifersharing.org). The UCSD IRB deemed this specific project an IRB exempt protocol given that the tissue was from a deceased individual and the research team obtained no donor identifiers.

### Experimental Procedures

Human diseased bicuspid valves were collected under an IRB approved protocol [19]. The control valves were obtained from organ donors whose hearts could not be used for transplantation. RNA was isolated from the valve leaflets using Trizol (15596, Invitrogen).

### Exposing Human Aortic Valve Interstitial Cells (AVICs) to Cyclic Stretch

Human aortic valve leaflets were harvested from organ donors whose hearts could not be transplanted under an IRB exempt protocol. Human AVICs were cultured according to standard protocols [41], [42]. Cells were grown on Collagen-1 coated Bioflex plates (BF-3001C Flexcell International). AVICs were exposed to cyclic stretch of 14% at 1 Hz using a Flexcell FX-5000 Tension system (Flexcell International) or static condition, the control condition, for 24 h at the same time on Bioflex plates.

### RNA Isolation

RNA was isolated using RNAeasy columns (74104, Qiagen) or Trizol (15596, Invitrogen) according to the manufacturer’s protocol.

### qPCR

cDNA was prepared using Superscript III (Invitrogen). qRT-PCR was performed using TaqMan primers (Applied Biosystems) for ALPL and BMP2 and SYBR Green primers for *HOTAIR*. ΔΔCT values were calculated; *GADPH* was used as the endogenous control. Student t-test was used to determine statistical significance. Standard error of the mean was presented as the error bars in the graphs representing the qPCR data.

### *HOTAIR* siRNA Treatment

AVICs were transfected with either *HOTAIR* siRNA [21] or scramble using lipofectamine [11], [19] for 96 h.

### DNA Microarray Experiments

Biotinylated cRNA was prepared using the Illumina RNA Amplification Kit, Catalog #1L1791 (Ambion, Inc., Austin, TX) according to the manufacturer’s directions starting with 250 ng total RNA. For microarray analysis, the Illumina HumanHT-12 v4 Expression BeadChip was used (Illumina, San Diego).

Hybridization of labeled cRNA to the BeadChip, and washing and scanning were performed according to the Illumina BeadStation 500x manual. Essentially the amplified, biotin-labeled mouse cRNA samples were resuspended in a solution of Hyb E1 buffer (Illumina) and 25% (v/v) formamide at a final concentration of 25 ng/µL. 1.5 µg of each cRNA were hybridized. Hybridization was allowed to proceed at 55°C, for 18 hours after which, the bead array matrix was washed for 10 minutes with 1X High temperature buffer (Illumina), followed by a subsequent 10 minute wash in Wash E1BC buffer. The arrays were then washed with 100% ethanol for 10 min to strip off any remaining adhesive on the chip. A 2-minute E1BC wash was performed to remove residual ethanol. The arrays were blocked for 5 minutes with 1% (w/v) casein-PBS, (Pierce). The array signal was developed via 10-minute incubation with Streptavidin-Cy3 at a final concentration of 1 µg/mL solution of (GE Healthcare) in 1% casein-PBS blocking solution. The Expression BeadChip was washed a final time in Wash E1BC buffer for five minutes and subsequently dried via centrifugation for 4 minutes at a setting of 275 rcf.

The arrays were scanned on the Illumina BeadArray Reader, a confocal-type imaging system with 532 (cye3) nm laser illuminations. Image analysis and data extraction was carried out as in accordance with Illumina specifications. Preliminary data analysis and QC was carried out using the GenomeStudio software (Illumina). All array data has been deposited in the EBI ArrayExpress Database (E-MTAB-2409).

### Analysis of Microarray Data

#### 1) Normalization of microarray data

Expression level data from the Illumina Bead Studio software were normalized using a quantile normalization [43]. Probes whose expression level exceeds a threshold value in at least one sample are called detected. The threshold value was found by inspection from the distribution plots of (log) expression levels.

#### 2) Sorting the probes according to significance

Detected probes were sorted according to their q-value, which is the smallest false discovery rate (FDR) at which the gene is called significant. FDR was the expected fraction of false positive tests among significant tests [36]. We evaluated FDR using Significance Analysis of Microarrays (SAM) and its implementation in the official statistical package samr [37]. In order to not be unduly impressed by accidentally small variances, we set the percentile of standard deviation values used for the exchangeability factor s0 in the test statistic to 75.

#### 3) Statistical analysis of pathways and gene ontology terms

Each gene ontology term or a pathway was treated simply as a set of genes. The probe list, sorted by q-value in ascending order, was translated into Entrez gene ID’s and parsed so that whenever several different probes represent the same gene, only the highest-ranking probe was kept for further analysis. The sorted list of genes was subjected to a non-parametric variant of the Gene Set Enrichment Analysis (GSEA) [38], in which the p-value of a gene set of size n was defined as follows: Let us denote the k-th highest rank in gene set as rk, and define pk as the probability that out of n randomly chosen ranks (without replacement) the k-th highest is not smaller than rk. The p-value of the gene set was defined as mink [Pk] It was designed to detect overrepresented gene sets at the top of the list. Unlike the Kolmogorov-Smirnov statistic used in GSEA, it would not detect underrepresented or other, pathologically distributed, gene sets. Finding the p-value of a gene set of size n required calculation of n rank-order values pk, however, there was no need to adjust the p-values for multiple testing as the rank-order tests are highly statistically dependent. We performed a Bonferroni adjustment of gene set p-values for the number of gene sets tested, even though there were often several gene sets with overlapping gene content (and therefore are statistically dependent), which was partly due to the design of the gene ontology database and partly because genes tended to be involved in multiple processes. We reported only gene sets with adjusted p-values ≤0.01.

### WNT Agonist Treatment

To examine the role of **β**-CATENIN signaling in AVICs, AVICs were treated with 10 µM of WNT agonist (2-Amino-4-(3,4-(methylenedioxy)benzylamino)-6-(3-methoxyphenyl)pyrimidine) [44] (Calbiochem) or DMSO (control) for 48 h.

## Results

### *HOTAIR* Levels are Decreased in BAVs and Stretched AVICs

**Figure 1:**
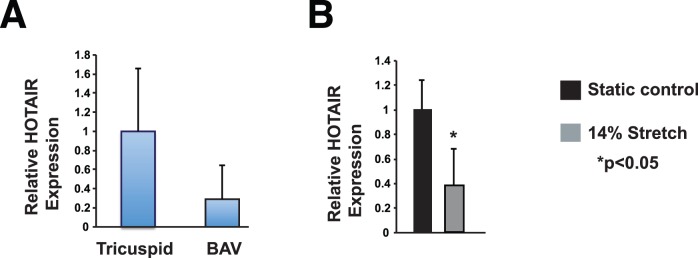
*HOTAIR* levels are decreased in BAVs and stretched AVICs. A. qPCR demonstrates that BAV leaflets have 69.3% lower levels of *HOTAIR* as compared to normal aortic valve leaflets (p = 0.08) n = 4. B. Cyclic stretch reduced *HOTAIR* levels by 67% (p<0.05) n = 3.

Since we had previously implicated microRNAs, small non-coding RNAs, to play a role in calcification of bicuspid aortic valves [19], we sought to examine whether lncRNAs are involved in modulating aortic valve calcification. *HOTAIR* was examined as a candidate lncRNA since it has a role in human disease [21], [22], [23], [24], [25]. To examine if *HOTAIR* levels are altered in diseased BAVs, *HOTAIR* qPCR was performed on BAV and control TAV leaflets. The BAVs have 69% less *HOTAIR* (p = 0.08) (Figure 1A). Since BAV leaflets experience increased strain, we examined if cyclic stretch was sufficient to modulate *HOTAIR* levels in AVICs. qPCR demonstrated that AVICs exposed to stretch have 67% decrease in *HOTAIR* levels (p<0.05) (Figure 1B).

### Cyclic Stretch Increases Expression of Calcification Related Genes

**Figure 2:**
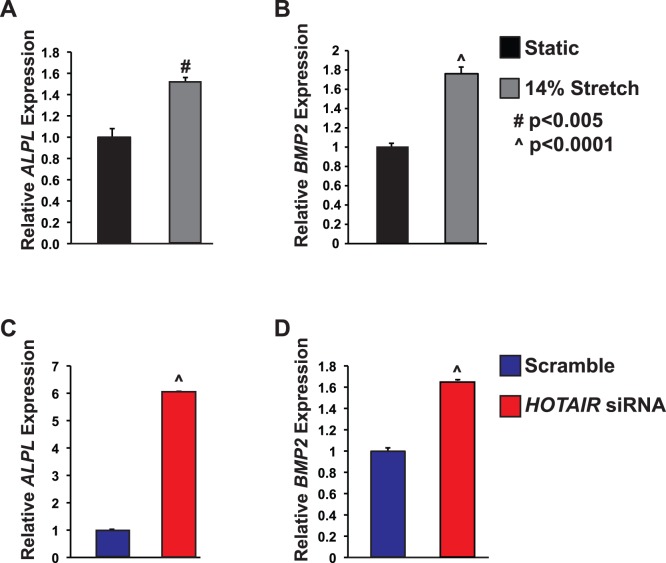
AVICs exposed to cyclic stretch or *HOTAIR* siRNA have increased expression of *ALPL* and *BMP2*. A and B. Human AVICs exposed to 14% stretch for 24 h have increased *ALPL* and *BMP2* mRNA levels, 50% and 77% respectively, as compared to static controls. N = 6. C and D. AVICs transfected with *HOTAIR* siRNA have more *ALPL* and *BMP2*, 6.06 fold, and 66% increase respectively, as compared to AVICs transfected with scramble control. N = 3. #P<0.005, ∧p<0.0001.

We had previously demonstrated that bicuspid aortic valve leaflets experience increased strain as compared to tricuspid aortic valves [8]; therefore, we sought to examine if cyclic stretch was sufficient to increased expression of *ALPL* and *BMP2*, two genes that are important in the calcification process. To model the increased strain experienced by AVICs in bicuspid aortic valves, we exposed human AVICs to cyclic stretch at 1 Hz for 24 h. In AVICs exposed to 14% cyclic stretch for 24 h had significantly increased expression of *ALPL* and *BMP2* as compared to static controls (Figure 2A and B). While *ALPL* has previously been found to be stretch responsive in AVICs [45], [46], *BMP2* had not previously been reported to be stretch responsive in AVICs.

### *HOTAIR* siRNA Activates Expression of Gene Related to Aortic Valve Calcification

Since *HOTAIR* levels were decreased by stretch and trended lower in bicuspid aortic valve leaflets, we examined if decreasing *HOTAIR* levels in AVICs resulted in alterations in *ALPL* and *BMP2* expression by using a previously published and validated siRNA [21]. *ALPL* and *BMP2* were identified as candidate *HOTAIR* target genes based upon examination of the supplemental data from Gupta et al [21]. qPCR demonstrated that AVICs transfected with *HOTAIR* siRNA had significantly increased expression of *ALPL* and *BMP2* (Figure 2C and D).

In order to examine if *HOTAIR* modulates additional genes and pathways associated with aortic valve calcification in an unbiased manner, we performed gene expression profiling comparing RNA from AVICs transfected with *HOTAIR* siRNA or scramble control. A number of aortic valve calcification associated genes were upregulated in *HOTAIR* siRNA samples (Table 1). Additionally, gene ontology (GO) terms such as inflammatory response (3.15×10–6), cytokine response (6.56×10–6), leukocyte migration (1.3×10−4), and ossification (2.05×10–2) were modulated by *HOTAIR* siRNA.

**Table 1:** Calcification Genes upregulated in *HOTAIR* siRNA microarray dataset.

Gene Symbol	Gene Name	Fold Change
BMP1	BONE MORPHOGENIC PROTEIN 1	1.31
BMP4	BONE MORPHOGENIC PROTEIN 4	1.36
BMP6	BONE MORPHOGENIC PROTEIN 6	1.81
BMPR2	BONE MORPHOGENIC PROTEIN RECEPTOR, TYPE II	1.34
COL1A1	COLLAGEN, TYPE 1, ALPHA 1	2.03
EDN1	ENDOTHELIN 1	1.69
MMP2	MATRIX METALLOPEPTIDASE 2	1.28
MMP10	MATRIX METALLOPEPTIDASE 10	1.24
MMP12	MATRIX METALLOPEPTIDASE 12	1.46
POSTN	PERIOSTIN	1.8

The full list of genes altered ≥1.2 fold with a p-value ≤0.05 is provided in Dataset S1. The biologic and molecular function GO terms modulated by *HOTAIR* siRNA are provided in Datasets S2 and S3.

### WNT Signaling is Sufficient to Repress *HOTAIR*

**Figure 3:**
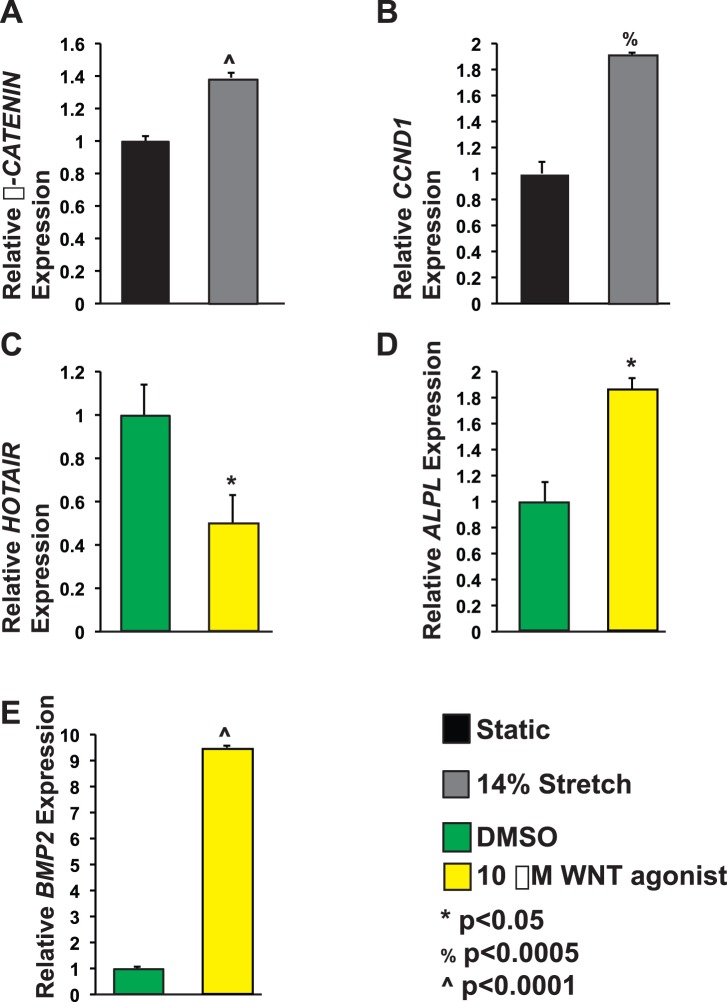
WNT/β-CATENIN signaling modulates *HOTAIR*, *ALPL*, and *BMP2* levels. A. AVICs exposed to cyclic stretch have 39% increased ***β*** *-CATENIN* mRNA levels as compared to static controls. N = 6. B. *Cyclin D1 (CCND1)* levels were examined in stretched AVICs since it is a known WNT**/β**-CATENIN target gene. Stretched AVICs have 91% increased *CCND1* expression as compared to static controls. N = 4. C. Treatment with WNT agonist is sufficient to repress *HOTAIR* by 50% as compared to DMSO treated controls. N = 3. D and E. AVICs treated with WNT agonist have increased *ALPL* and *BMP2*, 87% and 9.5 fold respectively. N = 3. *<p0.05, %p<0.0005, ∧p<0.0001.

Since WNT/**β**-CATENIN signaling has been implicated in aortic valve calcification [32], [33], [34], [35], we examined if WNT/**β**-CATENIN signaling plays a role in the stretch modulation of *ALPL*, *BMP2*, and *HOTAIR.* We performed qPCR for ***β*** *-CATENIN* and the **β**-CATENIN target gene *CYCLIND1* (*CCND1*) in stretched and static AVICs. We found that stretch increases expression of ***β*** *-CATENIN* and *CCND1* (Figure 3A and B).

To examine if WNT was sufficient to repress *HOTAIR*, we performed *HOTAIR* qPCR on RNA from the WNT agonist treated AVICs and found that WNT agonist treatment resulted in 50% less *HOTAIR* (Figure 3C). The **β**-CATENIN signaling mediated reduction in *HOTAIR* levels were also seen in AVICs treated with BIO, a GSK3**β** inhibitor that increases **β**-CATENIN signaling (Figure S1).

To examine if increased WNT/**β**-CATENIN signaling was sufficient to activate *ALPL* and *BMP2* in AVICs, we treated AVICs with a WNT agonist for 48 h and found that these AVICs had increased *ALPL* and *BMP2* mRNA levels as compared to controls (Figure 3D and E).

## Discussion

**Figure 4:**
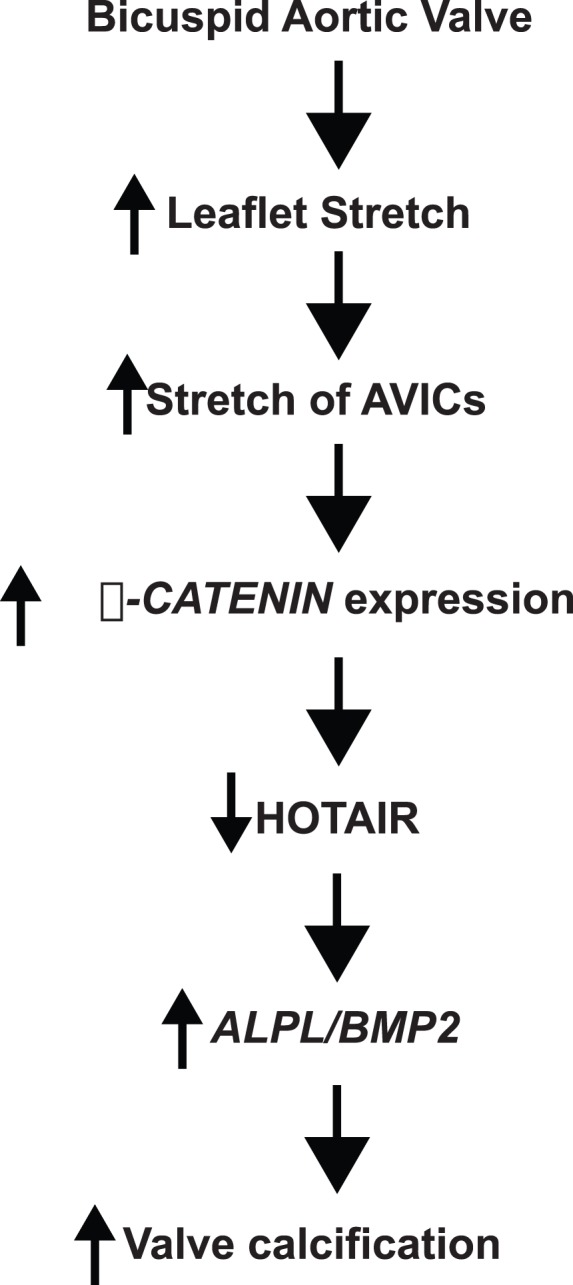
Proposed mechanism by which stretch-mediated repression of *HOTAIR* is involved in aortic valve calcification.

In this report, we demonstrate novel regulatory mechanisms for the lncRNA, *HOTAIR*, and implicate for the first time that *HOTAIR* plays a role in heart disease. We show that *HOTAIR* levels appear to be decreased in diseased BAVs as compared to TAVs. AVICs exposed to cyclic stretch have decreased *HOTAIR* levels. These data are the first report of biomechanical regulation of a lncRNA. Based upon our qPCR and microarray data, we demonstrate that *HOTAIR* represses *ALPL* and *BMP2* along with additional calcification and inflammatory genes. Stretch is sufficient to activate Wnt/**β**-CATENIN signaling in AVICs. Increasing Wnt signaling in AVICs results in increased calcification gene expression and decreased *HOTAIR* levels. As the result of these findings, we propose the model (Figure 4) that biomechanical stretch activation of calcification pathways in BAVs involves modulation of Wnt/**β**-CATENIN, *HOTAIR*, and epigenetic regulation of calcification related genes.

Our data finds that BAV leaflets tend to have lower *HOTAIR* levels as compared to normal TAVs. Since others and we have found that BAV leaflets experience increased strain [7], [8], our data that AVICs exposed to cyclic stretch have decreased *HOTAIR* are consistent with the biomechanical mechanism of BAV calcification. In addition to stretch-mediated repression of *HOTAIR* that appears to occur in BAVs, it is possible that SNPs and mutations in the *HOTAIR* locus could be associated with aortic valve calcification. Since currently very little is known about SNPs associated with aortic valve calcification [47], it is not possible answer this question with the current SNP data. Additionally, further study is required to examine if perturbation of *HOTAIR* is sufficient to induce aortic valve calcification *in vivo.*

While our finding that *HOTAIR* is repressed by biomechanical stretch, we anticipate that additional cardiac lncRNAs will be identified as being modulated by stretch. This belief is based upon the fact that the heart experiences stretch with each heart beat and the growing roles of lncRNAs. Additionally, the mechanisms by which biomechanical stretch regulate cardiac gene expression are especially poorly understood.

As the result of our data that stretch and WNT/**β**-CATENIN represses *HOTAIR* along with published data, we propose that cyclic stretch activates expression of *ALPL* and *BMP2* via the release of epigenetic repression. *HOTAIR* has been shown to recruit PRC2 complexes to trimethylate H3K27 [21], [26], [27], which silences transcription. Gupta et al identified potential *HOTAIR*/PRC2 binding sites by overexpressing *HOTAIR* in breast cancer cells and used antibodies to members of the PRC2 complex and H3K27me3 for ChIP-tiling array experiments [21]. Their published supplemental data demonstrated that increased *HOTAIR* expression resulted in increased binding of PRC2 complex to the *ALPL* and *BMP2* promoters. As a result of these data, we propose the model (Figure 4) that stretch increased WNT/**β**-CATENIN signaling which in turn represses *HOTAIR*, thereby decreasing H3K27 trimethylation at *ALPL* and *BMP2*. As a result of this release of epigenetic repression, stretched AVICs have increased expression of *ALPL, BMP2*, and other calcification related genes.

## Supporting Information

Figure S1 **Treatment with BIO is sufficient to repress** ***HOTAIR*** **in AVICs.** BIO is a potent activator of **β**-CATENIN signaling. AVICs treated with BIO have 56% less *HOTAIR* as compared to AVICs treated with meBIO, the negative control for BIO. N = 3. *p<0.05.
(EPS)

Dataset S1 **Excel file containing genes altered >1.2 fold with a p<0.05 in the** ***HOTAIR*** **siRNA microarray data.**
(XLS)

Dataset S2 **Excel file contain list of Biologic Process GO terms modulated by** ***HOTAIR*** **siRNA.**
(XLSX)

Dataset S3 **Excel file contain list of Molecular Function GO terms modulated by** ***HOTAIR*** **siRNA.**
(XLS)
