# Correction: The Long Non-Coding *HOTAIR* Is Modulated by Cyclic Stretch and WNT/β-CATENIN in Human Aortic Valve Cells and Is a Novel Repressor of Calcification Genes

**DOI:** 10.1371/journal.pone.0104370

**Published:** 2014-07-29

**Authors:** The PLOS ONE Staff

In Figures 3 and 4 of the published article, several mu (µ) and beta (β) symbols are represented by empty squares. Please view the correct figures below.

**Figure 3:**
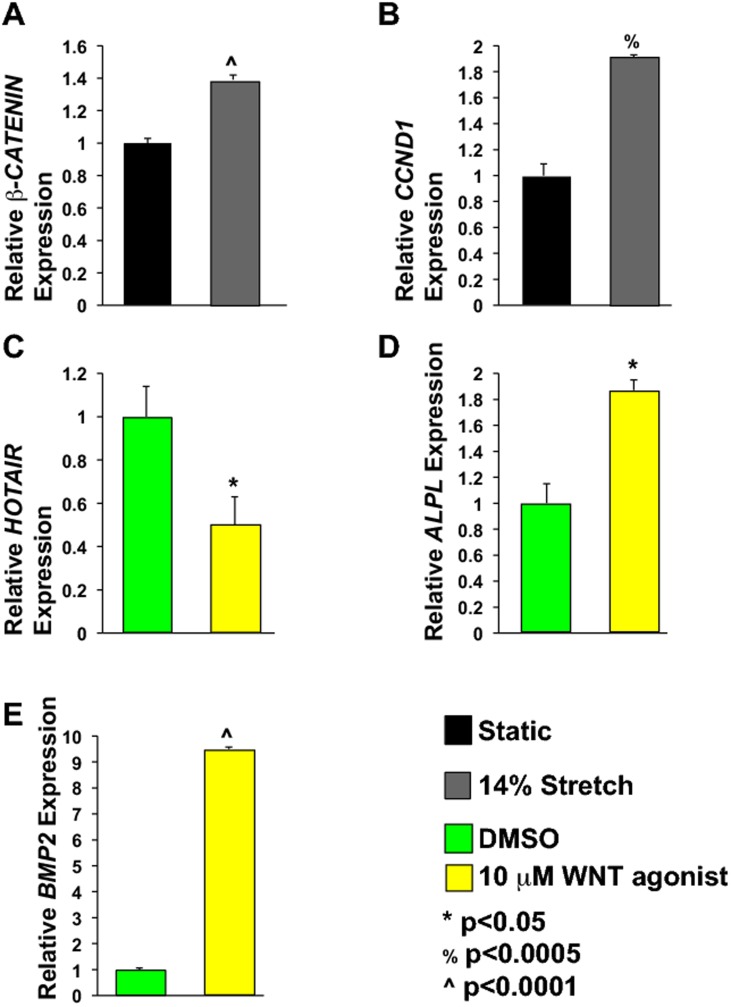
WNT/β-CATENIN signaling modulates *HOTAIR*, *ALPL*, and *BMP2* levels. A. AVICs exposed to cyclic stretch have 39% increased *β-CATENIN*mRNA levels as compared to static controls. N  =  6. B. *Cyclin D1 (CCND1)* levels were examined in stretched AVICs since it is a known WNT/β-CATENIN target gene. Stretched AVICs have 91% increased*CCND1* expression as compared to static controls. N  =  4. C. Treatment with WNT agonist is sufficient to repress *HOTAIR* by 50% as compared to DMSO treated controls. N  =  3. D and E. AVICs treated with WNT agonist have increased *ALPL* and *BMP2*, 87% and 9.5 fold respectively. N  =  3. *<p0.05, %p<0.0005, ∧p<0.0001.

**Figure 4:**
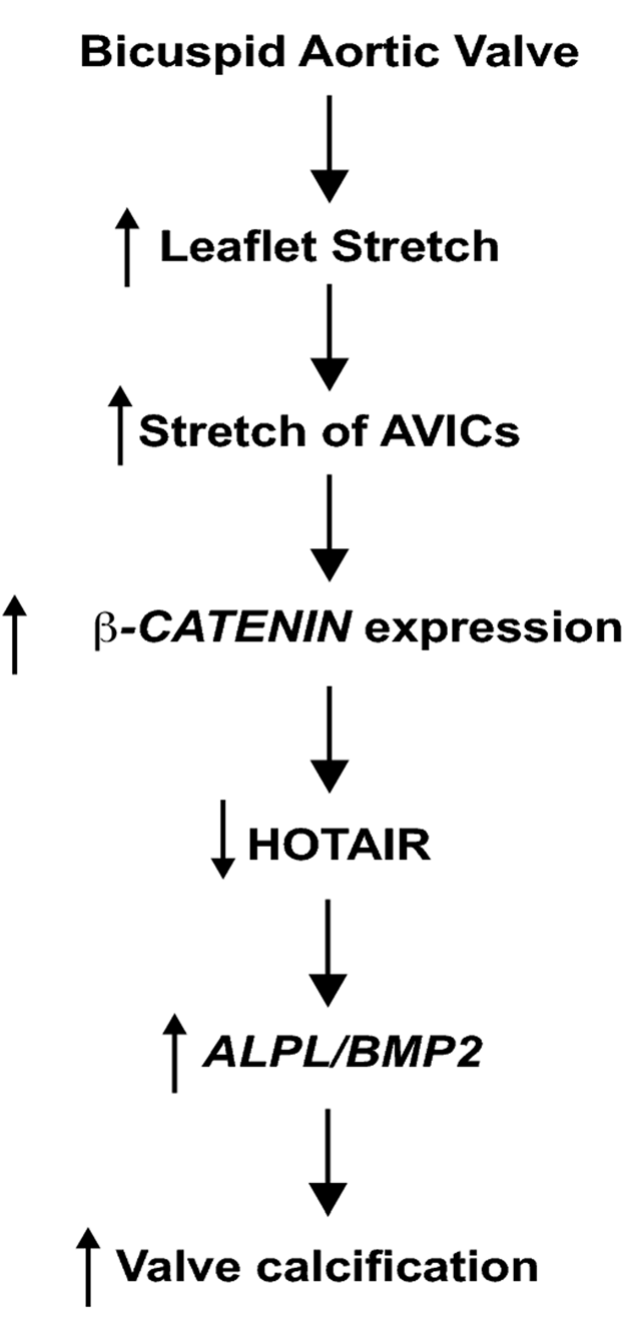
Proposed mechanism by which stretch-mediated repression of *HOTAIR* is involved in aortic valve calcification.
